# The asymmetrical ROS–METTL3–ESR1 axis in paraspinal muscle progenitor cells determines the progression of adolescent idiopathic scoliosis

**DOI:** 10.1038/s12276-026-01658-7

**Published:** 2026-03-05

**Authors:** Bin Li, Amila Kuati, Wenyuan Sui, Jinkui Cai, Tianyuan Zhang, Yaolong Deng, Junlin Yang, Xingzuan Lin, Xiexiang Shao

**Affiliations:** 1 Department of Spine Surgery, Xin Hua Hospital Affiliated to Shanghai Jiao Tong University School of Medicine, Shanghai, China; 2 Department of Rehabilitation and Sports Medicine, Peking University Third Hospital, Beijing, China; 3 Wuhan Third Hospital, Tongren Hospital of Wuhan University, Wuhan, China

**Keywords:** DNA methylation, Skeletal muscle

## Abstract

Adolescent idiopathic scoliosis (AIS) is the most common spinal deformity, yet its precise etiology remains elusive. Our previous research highlighted the pivotal role of the asymmetrical *ESR1* expression of paraspinal muscle stem/progenitor cells in the progression of AIS. However, the widespread distribution of ESR1 in various organs and tissues limits its safety and efficacy as a therapeutic target. Therefore, it is imperative to investigate the regulatory mechanisms governing the asymmetric *ESR1* expression in paraspinal muscle stem/progenitor cells to identify safer and more effective treatment strategies for AIS. Here we discovered elevated levels of reactive oxygen species (ROS) in the concave paraspinal muscles of patients with AIS. The increased ROS decreased the expression of m^6^A methyltransferase METTL3, which further diminished the expression of *ESR1* in an m^6^A-dependent manner in concave paraspinal muscle stem/progenitor cells. Thus, the asymmetrical ROS–METTL3–ESR1 axis in paraspinal muscle stem/progenitor cells plays a crucial role in the progression of AIS. Unilateral oxidative stress is one of the causes of AIS through the asymmetrical ROS–METTL3–ESR1 axis in paraspinal muscle stem cells. Furthermore, the antioxidant and methyl donor betaine effectively mitigated the differentiation defects of concave muscle stem/progenitor cells and alleviated the progression of scoliosis through targeting the ROS–METTL3–ESR1 axis. Reducing ROS and increasing *METTL3* expression in paraspinal muscle stem cells on the concave side may represent a novel therapeutic strategy for AIS.

## Reactive oxygen species influence muscle stem cells in scoliosis

Adolescent idiopathic scoliosis (AIS) is a common spinal condition. Researchers explored how certain cellular processes might influence AIS progression. They found that, in patients with AIS, the concave side of the spine’s muscles had higher levels of reactive oxygen species (ROS), which are harmful molecules. This increase in ROS reduced the activity of a gene-modifying enzyme called METTL3, leading to lower levels of ESR1, a protein important for muscle development. The study involved experiments on human muscle cells and mice to understand these changes. They discovered that high ROS levels impaired muscle cell growth by affecting METTL3 and ESR1. The researchers also tested betaine, a compound with antioxidant properties, which helped restore muscle cell function and reduced scoliosis progression in mice. The findings suggest that targeting ROS and enhancing METTL3 activity could be a new treatment strategy for AIS.

This summary was initially drafted using artificial intelligence, then revised and fact-checked by the author.

## Background

Adolescent idiopathic scoliosis (AIS) is the predominant form of three-dimensional spinal deformities with a lateral spine curvature of at least 10°, affecting approximately 0.5–5.2% of adolescents worldwide^1,2^. AIS is more prevalent in females (female-to-male ratio: 1.5:1–3:1) and tends to progress during the pubertal growth spurt^3,4^. Although patients with mild AIS can lead normal lives without serious complications, those with severe spinal deformities may suffer from chronic back pain, cardiorespiratory dysfunction and potentially life-threatening conditions^5–7^. A variety of factors have been suggested as contributors to AIS, including genetic, hormonal, musculoskeletal and environmental influences. However, the exact mechanism of AIS remains elusive^8^.

The spinal musculature has been proposed as an important factor in the progression of AIS since the 1970s^9^. The paravertebral muscles act as a pivotal role in maintaining spinal stability, and their imbalance is believed to contribute to spine biomechanical instability and result in the progression of a scoliotic curve in AIS^10^. Biopsies obtained from the bilateral paravertebral muscle show apparent pathological changes on the concave side of the spine, including type I fiber atrophy, fibrosis and fatty infiltration^11,12^. The asymmetry of these muscles has also been observed using magnetic resonance imaging, biomechanical tests, and electromyography, and has been reported to be associated with AIS^8,13,14^. However, while extensive research has focused on morphological studies, further investigation is needed to understand the underlying mechanisms leading to paravertebral muscle asymmetry.

Genome-wide association studies have identified *PAX3 and MYOD1*, which are pivotal transcription factors in muscle growth and regeneration, as susceptibility genes for AIS. Furthermore, studies have documented the asymmetric expression of *PAX3 and MYOD1* in patients with AIS^15–17^. Notably, our previous research revealed that asymmetrical *ESR1* expression in paravertebral muscles plays a crucial role in AIS progression and suggested a potential therapeutic approach using raloxifene, a US Food and Drug Administration-approved selective estrogen receptor modulator^18^. However, given that ESR1 is expressed in numerous tissues and organs, the paramount concerns are the safety and efficacy of raloxifene administration^19,20^. Consequently, it is imperative to delve deeper into the regulatory mechanisms governing the asymmetric *ESR1* expression in paraspinal muscle stem/progenitor cells to uncover safer and more effective treatment strategies for AIS.

As an important component of the epigenetic landscape, *N*^6^-methyladenine (m^6^A) stands out as the most prevalent internal modification on eukaryotic mRNA, exerting a pivotal influence on mRNA stability and the regulation of gene expression^21–23^. METTL3, a key m^6^A methyltransferase, is implicated in a multitude of biological processes, notably including the maintenance of muscle function^24–26^. Recent studies have shown that METTL3-mediated m^6^A modification regulates the myoblast transition from proliferation to differentiation^27^. Another study revealed that METTL3 is essential for stabilizing *MyoD* mRNA levels in myoblasts for skeletal muscle differentiation^28^. Overall, these results suggest METTL3 plays an important role in muscle progenitor cell proliferation and differentiation^29^.

In our study, elevated reactive oxygen species (ROS) in the concave paraspinal muscles were discovered in patients with AIS. The increased ROS decreased the expression of m^6^A methyltransferase METTL3, which further diminished the expression of *ESR1* in an m^6^A-dependent manner in concave paraspinal muscle stem/progenitor cells. Finally, decreased expression of *ESR1* caused defects in the differentiation of concave muscle stem/progenitor cells, exacerbating the severity of scoliosis. Furthermore, we used betaine (trimethyl glycine), a stable and nontoxic compound known for its potent antioxidant properties and its ability to enhance m^6^A methylation^30–34^, to effectively mitigate the differentiation defects of concave muscle stem/progenitor cells and alleviate the progression of scoliosis through targeting the ROS–METTL3–ESR1 axis. These findings elucidate the mechanism behind the asymmetric *ESR1* expression in paraspinal muscle stem/progenitor cells and suggest a safer and more viable therapeutic approach for AIS.

## Methods

### Animals

All experimental procedures were approved by the local institutional animal care and use committee (approval no. XHEC-F-2024-042). Female mice were used for all the animal experiment. The *Pax7-CreERT2* mice were sourced from The Jackson Laboratory (cat. no. 011763), while the *Mettl3* flox/flox mice were procured from Gem Pharmatech (cat. no. T006659). Muscle stem cell-specific gene knockout (KO) was achieved by administering intraperitoneal injections of 100 μl of a 10 mg/ml tamoxifen solution (ABCONE, cat. no. T56488) every 48 h for a period of 1 week. Bipedal mouse models were prepared as detailed in previous reports^35^. For the establishment of the scoliosis mouse model, 3-week-old bipedal mice received unilateral intramuscular injections of 100 μl 100 μM H_2_O_2_ (Sigma, cat. no. HX0640) at the left side and 100 μl phosphate-buffered saline (PBS) at the right side of the paraspinal muscle, which were carried out twice weekly for a duration of 3 weeks. In these oxidative-stress-induced scoliosis mouse models, intramuscular injections of 10 nM betaine (Vokai Biotechnology, cat. no. E11074) were administered to the concave paraspinal muscle twice weekly for 2 weeks. Assessments of spinal alignment and paraspinal muscle size were conducted 2 weeks after the final betaine injection.

### Human samples

Bilateral paraspinal muscles that were discarded during surgery at the level of the apical vertebra were collected as previously described^36^. The collection procedure posed no additional risk to the patients. The study was granted approval by the local institutional ethics committee (approval no. XHEC-D-2019-093), and written informed consent was obtained from all participants and, where applicable, their legal guardians.

### Isolation of muscle stem/progenitor cells

Muscle stem/progenitor cells were isolated according to previously established methods^18,37^. In summary, muscle tissues were sectioned and enzymatically dissociated using collagenase II and dispase (Worthington Biochemical, 700-800 U/ml, cat. no. LS004177; Life Technologies, 11 U/ml, cat. no. 17105-041). The resulting digest was passed through a 40-μm cell strainer (BD Falcon, cat. no. 352340). For human cells, the suspension was incubated with the following antibodies for 45 min at 4 °C: PE-Cy5 anti-human CD45 (BD Pharmingen, cat. no. 555484, diluted 1:25), PerCP-Cy5.5 anti-human CD31 (BioLegend, cat. no. 303132, diluted 1:100), AF-488 anti-human CD29 (BioLegend, cat. no. 303016, diluted 1:100), and BV421 anti-human CD56 (BD, cat. no. 562751, diluted 1:100). For mouse cells, the suspension was stained with APC anti-mouse CD31 (BioLegend, cat. no. 102510, diluted 1:100), APC anti-mouse CD45 (BioLegend, cat. no. 103112, diluted 1:100), FITC anti-mouse Sca1 (BioLegend, cat. no. 108106, diluted 1:100), and biotin anti-mouse VCAM1 (BioLegend, cat. no. 105703, diluted 1:100) for the same duration and temperature. All cell suspensions were washed with PBS and subsequently stained with PE/Cy7 streptavidin (BioLegend, cat. no. 405206, diluted 1:100) for 15 min. Finally, CD31^−^ CD45^−^ CD29^+^ CD56^+^ human muscle stem/progenitor cells and CD31^−^ CD45^−^ Sca1^−^ VCAM1^+^ murine muscle stem cells were obtained by BD Influx sorter (BD Biosciences)

### Cell culture and treatment

Primary human muscle stem/progenitor cells were cultured in F10 basal medium (Gibco, cat. no. 11550043) with 20% fetal bovine serum (Gibco, cat. no. 10-013-CV) and 2.5 ng/ml bFGF (R&D, cat. no. 233-FB-025). Mouse muscle stem cells were cultured in F10 basal medium (Gibco, cat. no. 11550043) with 20% fetal bovine serum (Gibco, cat. no. 10-013CV), 2.5 ng/ml bFGF (R&D, cat. no. 233-FB-025), 5 ng/ml IL-1α (Peprotech, cat. no. 211-11A), 5 ng/ml IL-13 (Peprotech, cat. no. 210-13), 5 ng/ml IFN-γ (Peprotech, cat. no. 315-05), 5 ng/ml TNF-α (Peprotech, cat. no. 315-01A) and 1% penicillin–streptomycin (Gibco, cat. no. 15140-122) in collagen-coated dishes at 37 °C in 5% CO_2_. The differentiation medium was Dulbecco’s modified Eagle medium (Gibco, cat. no. 11965118) with 2% horse serum (HyClone, cat. no. HYCLSH30074.03HI) and 1% penicillin–streptomycin (Gibco, cat. no. 15140-122). For the H_2_O_2_ treatment, the procedure was carried out as previously detailed. Muscle stem cells were subjected to 100 μmol/l H_2_O_2_ (Sigma, cat. no. HX0640) during myogenic differentiation. For betaine treatment, the human muscle stem/progenitor cells isolated from paravertebral muscles were treated with 10 nM betaine (Vokai Biotechnology, cat. no. E11074) during myogenic differentiation.

### RNA sequencing

Total RNA extraction from paraspinal muscle samples and stem/progenitor cells, collected from both the convex and concave regions, was performed utilizing the TRIzol reagent (Invitrogen). RNA libraries were constructed using the NEBNext Ultra RNA Library Prep Kit for Illumina (New England Biolabs, cat. no. E7530L). Next, a complementary DNA (cDNA) library was prepared using a nonstranded method. Paired-end sequencing was performed on a NovaSeq 6000 sequencer with a 2 × 150 bp read length. Subsequently, clean paired-end reads were aligned to the GRCh38.98 reference genome using HISAT2 (Hierarchical Indexing for Spliced Alignment of Transcriptomes, version 2), and gene abundances were quantified with RSEM (RNA-Seq by Expectation-Maximization) (http://deweylab.biostat.wisc.edu/rsem/). Gene Ontology (GO) analyses were performed using Goatools (https://github.com/tanghaibao/Goatools). The selection of the top GO categories was informed by the associated *P* values, which indicate the statistical significance of the identified gene functions and biological processes.

### RNA extraction and quantitative reverse transcription PCR (RT–qPCR)

Total RNA was isolated using TRIzol reagent (Invitrogen). Synthesis of cDNA was carried out using the PrimeScript Master Mix (TaKaRa), following the manufacturer’s protocol. Quantitative detection of mRNA expression was facilitated by the SYBR Premix Ex Taq II Kit (TaKaRa). The specific primer sequences utilized in this process are detailed in Table 1.

**Table 1:** The primers used in RT–qPCR.

Primer name	Forward (5′–3′)	Reverse (5′–3′)
Human *GAPDH*	CAAGGCTGAGAACGGGAAGC	AGGGGGCAGAGATGATGACC
Human *METTL*3	TTGTCTCCAACCTTCCGTAGT	CCAGATCAGAGAGGTGGTGTAG
Human *ESR1*	CCCACTCAACAGCGTGTCTC	CGTCGATTATCTGAATTTGGCCT
Human *MYH1*	GGGAGACCTAAAATTGGCTCAA	TTGCAGACCGCTCATTTCAAA
Human *MYH3*	ATTGCTTCGTGGTGGACTCAA	GGCCATGTCTTCGATCCTGTC
Human *CKM*	ATGCCATTCGGTAACACCCAC	GCTTCTTGTAGAGTTCAAGGGTC
Human *MYOG*	GGGGAAAACTACCTGCCTGTC	AGGCGCTCGATGTACTGGAT
Mouse *Gapdh*	ACCCAGAAGACTGTGGATGG	ACACATTGGGGGTAGGAACA
Mouse *Esr1*	CCCGCCTTCTACAGGTCTAAT	CTTTCTCGTTACTGCTGGACAG
Mouse *Myh1*	GCGAATCGAGGCTCAGAACAA	GTAGTTCCGCCTTCGGTCTTG
Mouse *Myh3*	ATGAGTAGCGACACCGAGATG	ACAAAGCAGTAGGTTTTGGCAT
Mouse *Ckm*	GGCAACACCCACAACAAGTTC	CCTTGAAGACCGTGTAGGACT
Mouse *MyoG*	GAGACATCCCCCTATTTCTACCA	GCTCAGTCCGCTCATAGCC

### Western blot analysis and protein extraction

Cells and tissues were lysed using RIPA buffer supplemented with protease inhibitors (Beyotime Biotech, cat. nos. P0013B and P1005). Protein concentrations were quantified by the bicinchoninic acid assay (Beyotime Biotech, cat. no. P0012). Protein expression levels were assessed via western blotting, as previously described. In brief, the proteins were then transferred onto polyvinylidene fluoride membranes (Millipore Sigma) after separated on sodium dodecyl sulfate–polyacrylamide gels and incubated overnight with primary and secondary antibodies. The primary antibodies used targeted ESR1 (Abcam, cat. no. A19665), METTL3 (Abcam, cat. no. ab195352, 1:1,000) and GAPDH (Cell Signaling Technology, cat. no. 2118S, 1:5,000). Chemiluminescent signals were visualized and photographed using the Classical ChemiDoc Imager (SHST).

### m^6^A sequencing and data analysis

m^6^A sequencing was conducted by CloudSeq Biotech. The raw sequencing reads were initially mapped to the reference mouse genome (mm10) using the Hisat2 software. Subsequently, the mapped reads from the immunoprecipitation (IP) and input libraries were analyzed using the R package exomePeak to identify pronounced m^6^A peaks and differential peaks, setting the significance threshold of the false discovery rate to ≤0.05. Finally, the Integrative Genomics Viewer software was used for visualization.

### Methylated RNA immunoprecipitation (MeRIP)–qPCR

The m^6^A RNA Methylation Fragment Enrichment Kit (Epigentek, P-9018) was used for the MeRIP studies, with all procedures strictly adhering to the manufacturer’s guidelines. In brief, 2 μg of RNA samples was set aside as the input control, while 18 μg of RNA samples, m^6^A antibody and affinity beads was mixed and then vortexed at room temperature for 90 min to facilitate m^6^A RNA immunocapture. Subsequently, a cleavage enzyme mix was used to generate RNA fragments. Proteinase K and an RNA purification solution were added to remove excess proteins and isolate m^6^A-enriched RNA. Ultimately, the immunoprecipitated m^6^A RNA was obtained using elution buffer. The m^6^A enrichment in ESR1 mRNA was quantified by RT–qPCR and normalized to the input levels. The specific primers used for screening are presented in Table 2.

**Table 2:** The primer sequences of five specific primers of ESR1 for MeRIP–qPCR.

Primer name	Forward (5′–3′)	Reverse (5′–3′)
*Esr1-Seg1*	TTCTGACAATCGACGCCAGAA	TCTTAAAGAAAGCCTTGCAGCC
*Esr1-Seg2*	GATAAGCACTTCATAATGGCTCCA	CATGTTGCTATAGGAATGCAAGC
*Esr1-Seg3*	GTCACAATGAACCTGCAAGC	ATTCTCCACATTTCTCCCTTACT
*Esr1-Seg4*	GAGTCCTTTGAACAAGGGGAT	CCCATCATATCTCAATGGAGTTC
*Esr1-Seg5*	TAGCTAATGGGTCAGTGGGTTCT	AGATGGGATAATGTAAAACCCTCC

### Luciferase reporter assay

The luciferase reporter assay was applied to find the functional methylation site in *ESR1*. The pmiRGLO vector (MIAOLING BIOLOGY, P0198) was used as the plasmid vector. The wild-type (WT) 3′ untranslated region (UTR) of *ESR1* (*ESR1*-3′UTR-WT) or mutant 3′ UTR of *ESR1* (*ESR1*-3′UTR-Mut) was inserted behind the F-luc coding region, respectively. The transfection of pmiRGLO-*ESR1*-3′UTR-WT and pmiRGLO-*ESR1*-3′UTR-Mut (A-to-G mutation at position 2,409) into cells was facilitated using Lipo3000 reagent (Invitrogen). Subsequent luciferase assays were carried out with the Dual-Luciferase Reporter Assay Kit (Yeasen Biotechnology, 11402ES60)), following the protocol provided by the kit.

### RNA and protein stability analysis

To perform RNA stability assay, actinomycin D (MCE, cat. no. HY-17559, 5 μg/ml) was added to inhibit the mRNA transcription of muscle stem/progenitor cells for 0, 3, 6, 9 and 12 h (ref. ^38^). The total RNA was collected and reverse-transcribed into cDNA. The *ESR1* RNA levels were determined using RT–qPCR, and the RNA degradation rate was calculated with *GAPDH* serving as the normalization reference. To evaluate protein stability, muscle stem/progenitor cells were treated with cycloheximide (MCE, cat. no. HY-12302, 100 μg/ml) to inhibit protein synthesis. Then, the total protein was collected at specific time points at 0, 3, 6, 9 and 12 h for subsequent analysis. ESR1 and GAPDH protein expression was assessed using western blotting^39^.

### Measurement of ROS levels

The assessment of total ROS levels was conducted using the ROS detection assay kit (BestBio, China, cat. no. BB-460512). In brief, the homogenate was prepared from 20 mg of fresh paravertebral muscle tissue from patients with AIS and centrifuged at 4 °C for 10 min. Then, 190 μl of supernatant was collected and incubated with 10 μl of BBcellProbeTM O11 ROS probe in a 96-well plate at 37 °C in the dark for 45 min. A multifunction microplate reader (Multiskan GO, Thermo Scientific) was used to measure ROS levels at an excitation/emission wavelength of 488/530 nm.

### Global RNA m^6^A content quantification

The global m^6^A modification level in total RNA was determined following the protocol of the m^6^A RNA Methylation Quantification Kit (MEIMIAN, cat. no. MM-2109H1). In summary, 200 ng of RNA was aliquoted into the assay wells, followed by the addition of the appropriately diluted detection antibody solution. The m^6^A levels were then quantified through colorimetric analysis by measuring the absorbance at 450 nm and correlating the results with the standard curve provided.

### Immunohistology and immunofluorescent staining

Fresh muscle tissues were embedded in optimal cutting temperature compound (OCT) and sectioned using a cryostat (Leica, cat. no. CM1860) to produce 10-μm-thick slices. Both the tissue sections and cultured cells were fixed with 4% paraformaldehyde (Sigma-Aldrich, cat. no. 30525) for 15 min and permeabilized with 0.1% Triton X-100 for 10 min at room temperature. Following this, they were blocked with 1% bovine serum albumin (Beyotime Biotechnology, cat. no. ST023). Subsequently, the sections and cells were incubated with anti-Laminin (Abcam, cat. no. ab11575, diluted 1:500) or anti-MyHC (Millipore, cat. no. 05-716, diluted 1:1,000) primary antibodies overnight. Alexa 488 or Alexa 594-conjugated anti-mouse or anti-rabbit secondary antibodies (Invitrogen, cat. nos. A11034 and A11005, diluted 1:1,000) were applied to visualize the target protein. 4′,6-Diamidino-2-phenylindole (DAPI, Vector Laboratories, cat. no. H-1200) was used for nuclear counterstaining, and the samples were finally mounted using an antifluorescence mounting medium (Vector Laboratories, cat. no. H-1200).

### Measurement of myofibers and myotubes

A minimum of five random visual fields were randomly selected and assessed for each sample. Laminin staining delineated the boundaries of myofibers, while MyHC staining outlined the contours of myotubes. The ImageJ software was used to enumerate cell nuclei (both total cell nuclei and those within myotubes) and to measure the cross-sectional area of the myofibers. All imaging analyses and evaluations were performed by investigators in a blinded manner to ensure objectivity.

### X-ray assessment

X-ray assessment was performed for anesthetized mice to evaluate spinal alignment as described in previous reports^35^. Radiographic images in both coronal and sagittal planes were captured using the Faxitron X-ray specimen radiography system (MX-20). The images were independently evaluated by two certified spine surgeons who were blinded to the study conditions, focusing on measuring the Cobb angles in the coronal and sagittal plane.

### Statistical analysis

All experiments were performed at least three times to ensure reproducibility. Data points and error bars depicted in the graphs represent the mean values ± standard deviation. Statistical comparisons between groups were made using a two-tailed Student’s *t*-test, as implemented in GraphPad Prism 7 software. A *P* value less than 0.05 was considered to indicate statistical significance. *P < 0.05, **P < 0.01, ***P < 0.001; ‘ns’ indicates no significant changes.

## Results

### Muscle stem/progenitor cells on the concave side of patients with AIS are exposed to high ROS levels, accompanied by suppressed METTL3 expression

**Fig. 1:**
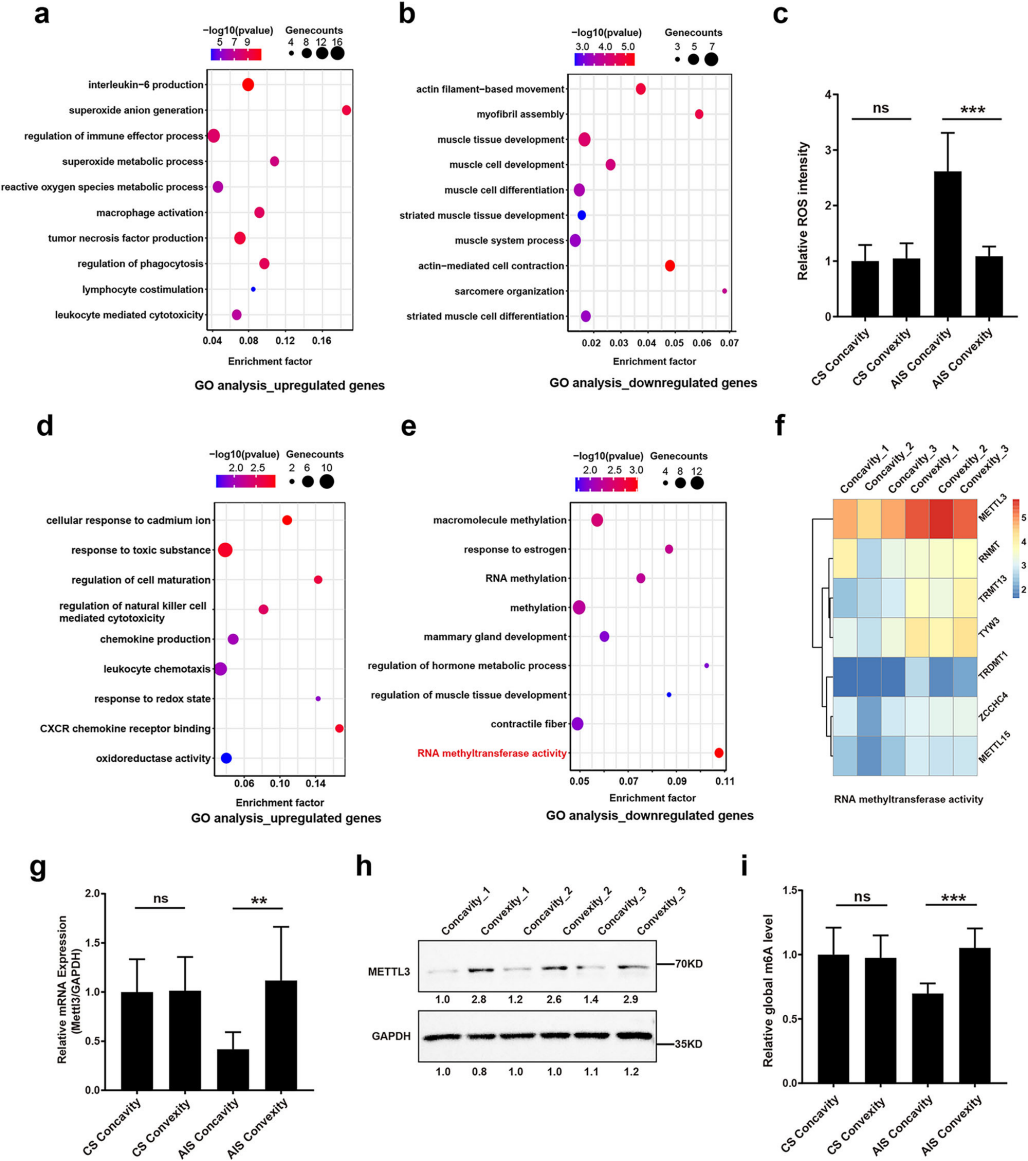
Muscle stem/progenitor cells on the concave side of patients with AIS are exposed to high ROS levels and exhibit suppressed METTL3 expression. **a**,**b** Bubble chart of GO analysis of upregulated (**a**) and downregulated (**b**) genes in paraspinal muscle tissues collected from the concave side compared with those from the convex side in patients with AIS (*n* = 3). **c** Relative ROS intensity in bilateral paraspinal muscles derived from patients with CS (*n* = 10) and AIS (*n* = 9). ***P < 0.001; ns indicates no significant changes. **d**,**e** Bubble chart of GO analysis of upregulated (**d**) and downregulated (**e**) genes in muscle stem/progenitor cells collected from concave paraspinal muscles compared those from convex paraspinal muscles in patients with AIS. **f** Heat map representation of downregulated genes in concave paraspinal muscle stem/progenitor cells in GO term RNA methyltransferase activity. **g** Relative mRNA expression of *METTL3* in bilateral muscle stem/progenitor cells collected from the patients with CS (*n* = 10) and AIS (*n* = 9). **P < 0.01; ns indicates no significant changes. **h** The protein level of METTL3 of bilateral muscle stem/progenitor cells collected from patients with AIS. GAPDH served as the internal reference. **i** The relative global m^6^A levels in bilateral muscle stem/progenitor cells collected from the patients with CS (*n* = 10) and AIS (*n* = 9). **P < 0.01; ns indicates no significant changes.

To analyze the features of bilateral paraspinal muscles, we first conducted RNA sequencing (RNA-seq) analysis. The GO enrichment analysis demonstrated heightened expression of genes associated with inflammation and ROS, as well as diminished expression of genes related to muscle function and development, specifically on the concave side of patients with AIS (Fig. 1a,b). Subsequent measurements of ROS levels in bilateral paraspinal muscles also validated the RNA-seq findings, showing a 140.8% increase in ROS levels in the concave paraspinal muscle compared with the convex side in patients with AIS, whereas no difference was observed between bilateral paraspinal muscles in patients with congenital scoliosis (CS) (Fig. 1c). These initial findings suggested that muscle stem/progenitor cells on the concave side of patients with AIS were subjected to oxidative stress. To further elucidate the expression profile difference of bilateral muscle stem/progenitor cells, we performed and analyzed RNA-seq on these cells. Consistently, GO enrichment analysis also revealed an increase in terms related to inflammatory infiltration and redox reactions (Fig. 1d). In addition, it is worth noting that downregulated genes were enriched for terms associated with RNA methyltransferase activity (Fig. 1e), with the key m^6^A methyltransferase *METTL3* identified as one of the markedly downregulated genes within the GO term ‘RNA methyltransferase activity’ (Fig. 1f). We subsequently corroborated the RNA-seq results using RT–qPCR and western blot analyses at both the mRNA and protein levels, which consistently showed decreased METTL3 expression in muscle stem/progenitor cells on the concave side of patients with AIS (Fig. 1g,h). By contrast, no such variation in METTL3 was observed in paraspinal muscle stem/progenitor cells from control participants (patients with CS) (Fig. 1g). Because METTL3 acts as a major writer of m^6^A modification, we then assessed the global m^6^A methylation levels in muscle stem/progenitor cells from both sides. The results demonstrated a reduction in global m^6^A levels in cells from the concave side compared with the convex side, while paraspinal muscle stem/progenitor cells from patients with CS did not exhibit asymmetric m^6^A levels (Fig. 1i). Collectively, these findings underscore that muscle stem/progenitor cells on the concave side of patients with AIS are exposed to high ROS levels accompanied by suppressed METTL3 expression.

### High levels of ROS could impair the myogenesis of muscle stem/progenitor cells by suppressing the expression of METTL3

**Fig. 2:**
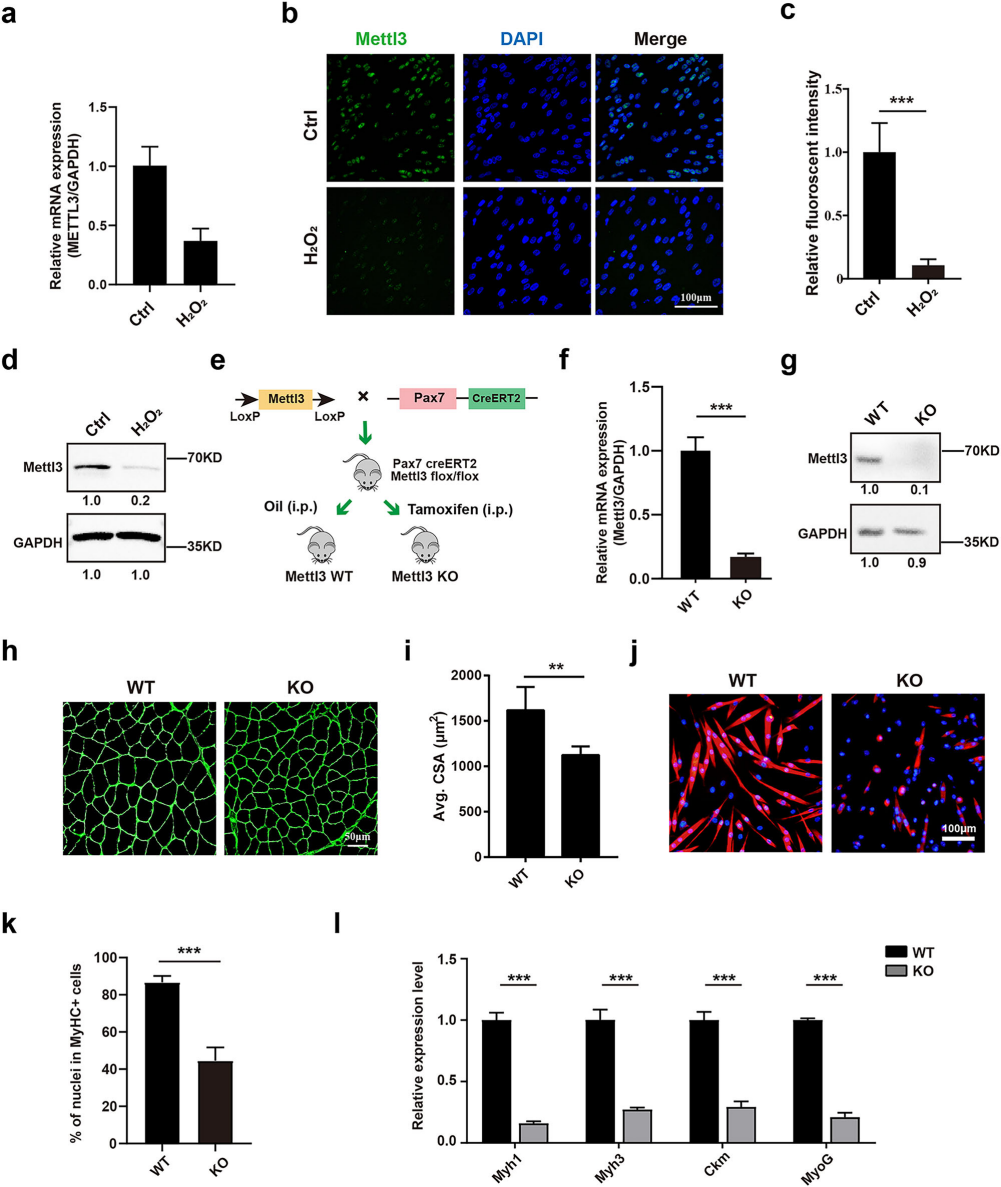
High levels of ROS could impair the myogenesis of muscle stem/progenitor cells by suppressing the expression of METTL3. **a** Relative mRNA expression of *METTL3* in human primary muscle stem/progenitor cells treated with or without H_2_O_2_. **b**,**c** Representative immunofluorescent staining (**b**) and relative statistical analysis (**c**) of human primary muscle stem/progenitor cells treated with or without H_2_O_2_. Green indicates METTL3; blue indicates DAPI staining of nuclei. The merged images are shown. Scale bars, 100 μm. ***P < 0.001. **d** The protein level of METTL3 and ESR1 in human primary muscle stem/progenitor cells treated with or without H_2_O_2_. GAPDH served as the internal reference. **e** Diagram illustrating the generation of *Pax7-CreERT2*; *Mettl3* flox/flox mice. **f**,**g** The relative mRNA (**f**) and protein (**g**) expression level of Mettl3 in muscle stem cell isolated from *Mettl3* flox/flox mice (WT) and *Pax7-CreERT2*; *Mettl3* flox/flox mice (KO). **h** Representative immunofluorescent staining of paraspinal muscle cryosections from *Mettl3* WT and KO mice. Green indicates laminin. Scale bars, 50 μm. **i** Statistical analysis of cross-sectional area of paraspinal muscle derived from *Mettl3* WT and KO mice, respectively. At least 500 fibers were analyzed for each sample. *n* = 5. **P < 0.01. **j** Representative immunofluorescent staining of myotubes differentiated from muscle stem cells isolated from *Mettl3* WT and KO mice. Red indicates MyHC; blue indicates DAPI staining of nuclei. The merged images are shown. Scale bars, 100 μm. **k** Quantification of the percentage of nuclei in MyHC^+^ cells. ***P < 0.001. **l** Relative mRNA expression levels of myogenic differentiation markers. Total RNA was extracted from myotubes differentiated from muscle stem cells isolated from WT and KO mice, and then RT–qPCR analysis was performed. *n* = 3. ***P < 0.001.

Based on the finding that muscle stem/progenitor cells on the concave side of patients with AIS are exposed to high ROS levels with decreased METTL3, we hypothesized that elevated ROS activity may suppress METTL3 expression, consequently impairing the differentiation of these cells. To test this hypothesis, we first established a high-ROS cell model using 100 μM H_2_O_2_ to investigate its effects on freshly isolated human primary muscle stem/progenitor cells from paraspinal muscles. Following treatment with H_2_O_2_, the expression of METTL3 was considerably decreased in both mRNA and protein levels tested by RT–qPCR, immunofluorescent staining and western blot tests (Fig. 2a–d). Thus, high levels of ROS markedly suppressed the expression of METTL3 in muscle stem/progenitor cells. To further explore the potential role of Mettl3 in myogenesis of muscle stem/progenitor cells, we generated muscle stem cell-specific *Mettl3*-KO mice by administering intraperitoneal tamoxifen injections to 3-week-old *Pax7-CreERT2*; *Mettl3* flox/flox mice (Fig. 2e). The isolation of muscle stem cells was carried out as previously described, and the KO efficiency of Mettl3 was confirmed through RT–qPCR and western blot tests (Fig. 2f,g). The paraspinal myofiber size in *Mettl3*-KO mice was observed to be smaller compared with that of WT mice at 8 weeks old (Fig. 2h,i). We then investigated the role of Mettl3 in myogenic differentiation. Muscle stem cells derived from *Mettl3*-WT and *Mettl3*-KO mice were induced to differentiate for 48 h. The immunofluorescent staining of MyHC revealed a decrease in myotube size and differentiation efficiency in *Mettl3*-KO cells (Fig. 2j,k). Consistently, the expression of differentiation markers, including *Myh1*, *Myh3*, *Ckm* and *MyoG*, was also found to be decreased (Fig. 2l).

To further investigate whether the ROS–METTL3–ESR1 axis drives asymmetry, METTL3 was overexpressed in convex-side progenitor cells. METTL3-overexpressing plasmid was transfected in convex human muscle stem/progenitor cells, while empty plasmid was transfected in bilateral human muscle stem/progenitor cells as a control. Western blot tests revealed increased ESR1 protein levels after METTL3 overexpression in convex-side progenitors (Supplementary Fig. 1a). The immunofluorescent staining of MyHC showed more MyHC-positive myotubes in convex muscle progenitor cells than concave muscle progenitor cells, while overexpression of METTL3 in convex muscle progenitor cells further increased the myogenic differentiation ability (Supplementary Fig. 1b,c). In addition, RT–qPCR analysis of myogenic differentiation markers *MYH1*, *MYH3*, *CKM* and *MYOG* showed that METTL3 overexpression in convex-side progenitor cells could induce asymmetrical differentiation (Supplementary Fig. 1d).

Together, these results suggest that high levels of ROS lead to a decrease in METTL3 expression in muscle stem/progenitor cells, further impairing the myogenic differentiation ability.

### Mettl3-mediated m^6^A modification regulates *Esr1* mRNA stability

**Fig. 3:**
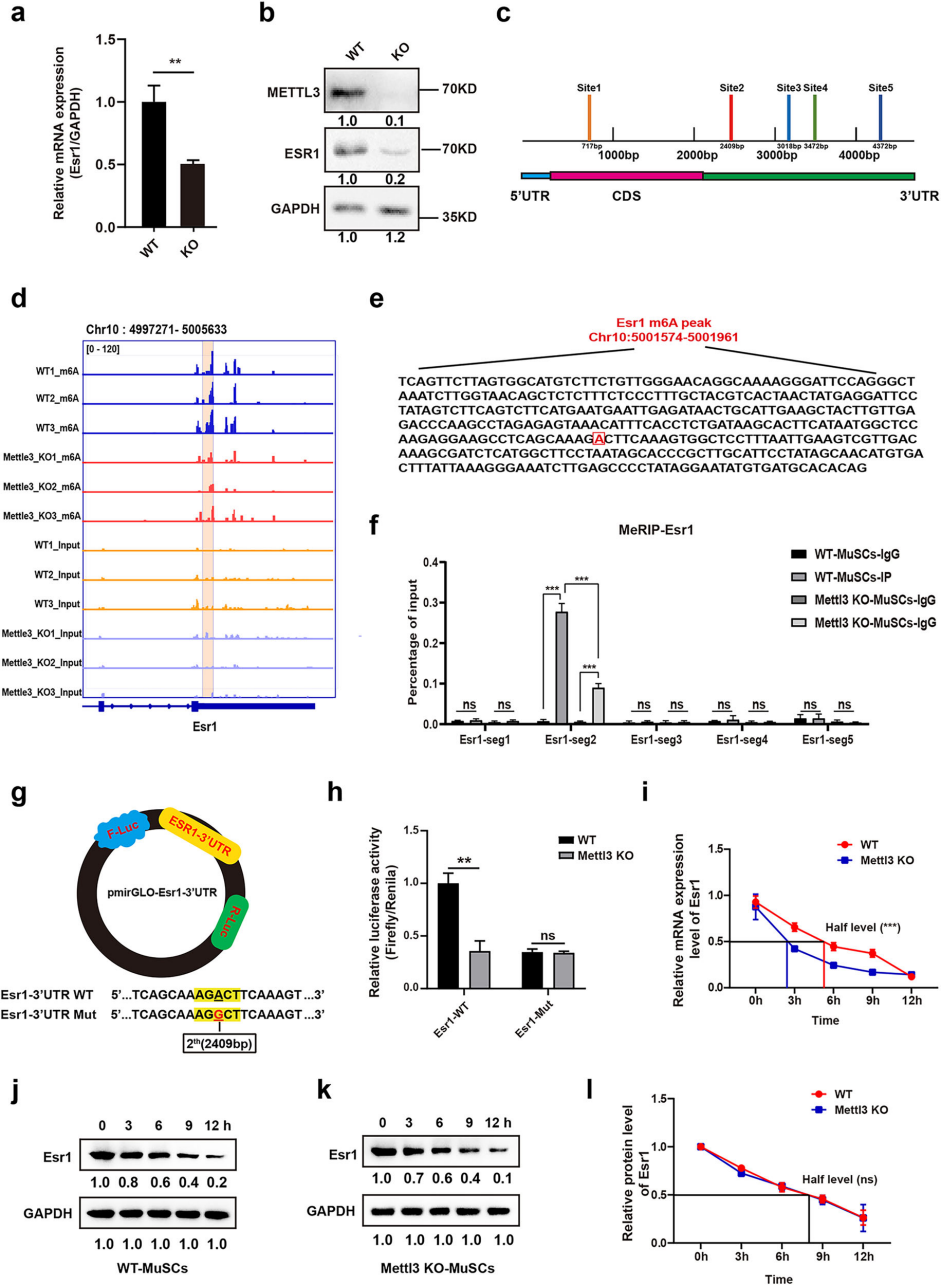
Mettl3-mediated m^6^A modification regulates *Esr1* mRNA stability. **a** Relative mRNA expression of *Esr1* in muscle stem cells isolated from *Pax7-CreERT2*; *Mettl3* flox/flox (WT) and *Pax7-CreERT2*; *Mettl3* KO (KO) mice. **P < 0.01. **b** The protein level of Esr1 and Mettl3 in muscle stem cells isolated from WT and KO mice. **c** Potential m^6^A modification sites on *Esr1* mRNA predicted by SRAMP. **d** MeRIP-seq showing m^6^A peak results of *Esr1* in *Mettl3* flox/flox and *Mettl3*-KO MuSCs. **e** Detailed gene sequence about the m^6^A peak of *Esr1* located at chromosome 10: 5001574–5001961. The predicted functional m^6^a modification site 2,409-bp A (highlighted) was also located in this gene sequence. **f** MeRIP–qPCR experiment of five segments on *Esr1*. ***P < 0.001. **g** Schematic representation of luciferase reporter assays. WT *Esr1*-3′UTR, or mutant of the second potential site (Mut: AGACT to AGGCT) *Esr1*-3′UTR was individually inserted behind the F-luc coding region in the luciferase reporter. **h** Rrelative luciferase activity. **P < 0.01; ns indicates no significant changes. **i** Results of mRNA stability assay. *Esr1* mRNA level were determined by RT–qPCR in muscle stem cells from WT and KO mice after actinomycin D treatment (normalized to 0 h). ***P < 0.001. **j**–**l** Results of protein stability assay. Esr1 protein levels were determined by western blot tests in muscle stem cells from WT (**j**) and KO (**k**) mice after cycloheximide treatment (normalized to 0 h) (**l**).

Mettl3 plays a notable role in post-transcriptional regulation through m^6^A modification. Building on our previous research highlighting the critical role of asymmetrical *ESR1* expression in the progression of patients with AIS, we further investigated the functional relationship between METTL3 and ESR1. We assessed Esr1 levels in muscle stem cells from Mettl3-KO mice using RT–qPCR and western blot tests, which revealed a marked reduction in Esr1 levels at both the mRNA and protein levels (Fig. 3a,b). These data indicate that Mettl3 can regulate *Esr1* expression in an m^6^A-dependent manner.

To characterize the potential mechanism of how m^6^A methylation regulated *Esr1*, we first conducted a SRAMP (sequence-based RNA adenosine methylation site predictor) analysis (http://www.cuilab.cn/sramp/) to identify potential m^6^A modification loci on *Esr1*^40^. The analysis indicated the presence of five possible m^6^A modification loci along the full length of *Esr1* (Fig. 3c). Subsequently, we conducted MeRIP-seq analysis on *Mettl3*-KO and *Mettl3*-WT muscle stem cells (MuSCs). The m^6^A methylation peak calling was obtained using the algorithm exomePeak. The results show that *Esr1* was modified by m^6^A methylation, with a marked decrease in m^6^A enrichment in the region of chromosome 10 from positions 4997271 to 5005633 (Fig. 3d,e). The predicted functional m^6^A modification site 2, the 2,409-bp A was also located in this region (Fig. 3c). To further validate the effective m^6^A modification segments within *Esr1*, we designed five specific primer pairs to amplify discrete regions corresponding to five predicted loci and performed MeRIP–qPCR tests. The results indicated that *Esr1*-seg2 (including the second potential m^6^A methylation sites located in the 3′ UTR region of *Esr1*) displayed a high level of m^6^A methylation in WT MuSCs, while showing a marked decrease in m^6^A levels on *Esr1* in *Mettl3*-KO MuSCs, supporting that *Esr1* could be a target for Mettl3-mediated m^6^A methylation in MuSCs (Fig. 3f). To verify the potential m^6^A modification site (2,409 bp), we used luciferase reporter assays. The WT *Esr1*-3′UTR and mutant (Mut, A-to-G mutation at position 2,409 bp) *Esr1*-3′UTR were inserted behind the F-luc coding region in the luciferase reporter plasmids (Fig. 3g). The luciferase activity is obviously lower in *Mettl3*-KO MuSCs than in WT MuSCs when transfected with *Esr1*-WT plasmid. There was no luciferase activity difference between WT and *Mettl*-KO MuSCs when transfected with *Esr1*-3′UTR Mut plasmid, suggesting that the second loci of *Esr1* acted as the effective site governing *Esr1* m^6^A modifications (Fig. 3h). To further investigate the impact of Mettl3-mediated m^6^A modification on *Esr1* expression, an mRNA stability test was performed, with the results showing that KO of *Mettl3* notably reduced the stability of *Esr1* mRNA after actinomycin D treatment (Fig. 3i). In addition, protein stability assays revealed that *Mettl3* KO did not affect the protein stability of Esr1 (Fig. 3j–l). These results indicate that Mettl3 regulates *Esr1* expression by stabilizing its mRNA at the post-transcriptional level.

In summary, these data indicate that Mettl3-mediated m^6^A modification could regulate *Esr1* mRNA stability and ultimately affect *Esr1* expression.

### Unilateral oxidative stress of paraspinal muscle leads to scoliosis through ROS–Mettl3–Esr1

**Fig. 4:**
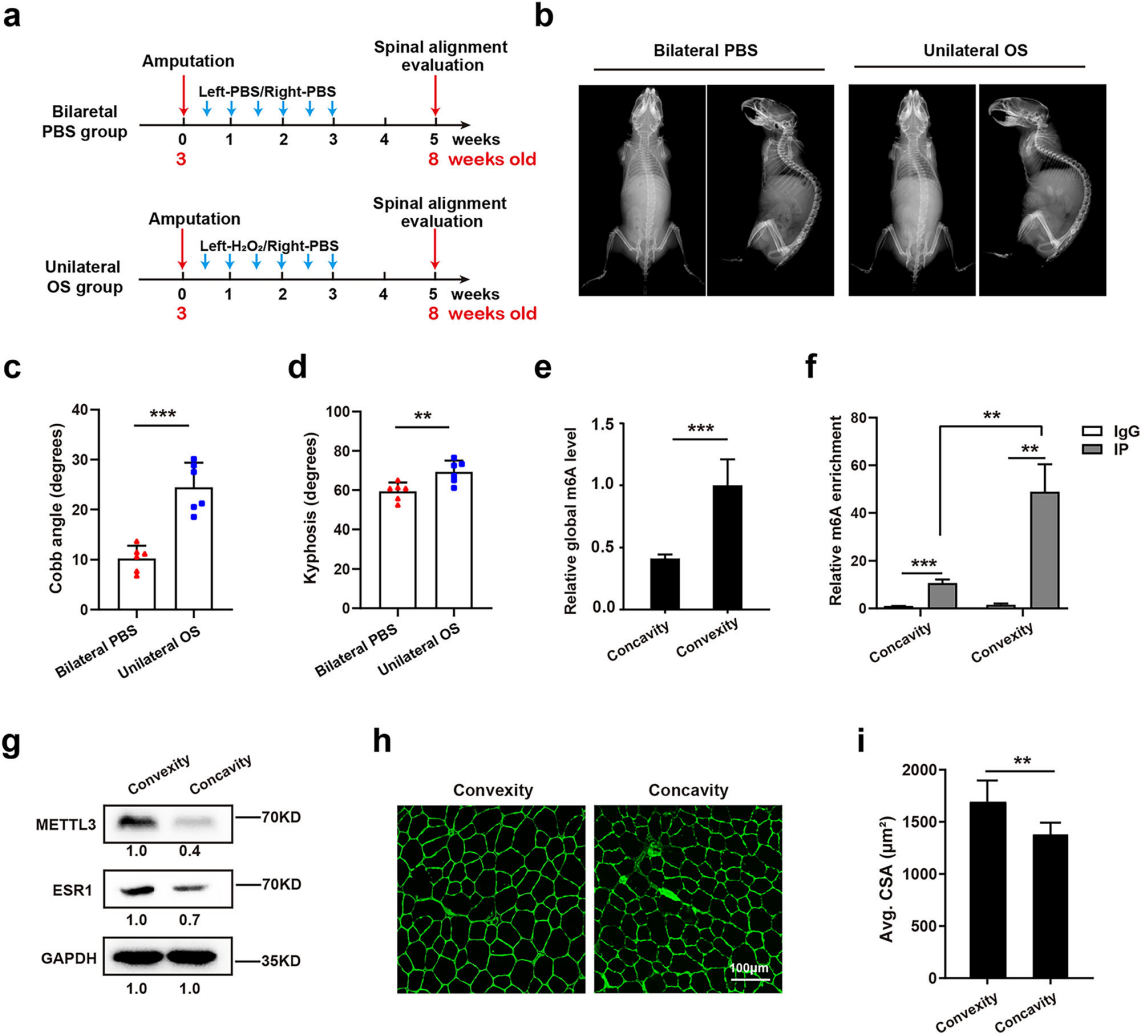
Unilateral oxidative stress of paraspinal muscle leads to scoliosis through the ROS–Mettl3–Esr1 axis. **a** Diagram of unilateral oxidative stress (OS) treatment in a bipedal mouse model. The spinal alignment was evaluated 5 weeks after the first injection. **b** X-ray images for spinal alignment in bilateral PBS and unilateral OS group 5 weeks after the first injection. **c**,**d** Statistical analysis of Cobb angle and kyphosis in coronal and sagittal plane for bilateral PBS group (*n* = 6) and unilateral OS group (*n* = 6). **P < 0.01, ***P < 0.001. **e** The relative global m^6^A level in bilateral muscle stem/progenitor cells collected from muscle tissues harvested from the concave and convex paraspinal muscles in unilateral OS mice. ***P < 0.001. **f** MeRIP–qPCR results for target m^6^A modification site in bilateral muscle stem/progenitor cells collected from muscle tissues harvested from the concave and convex paraspinal muscles in unilateral OS mice. **P < 0.01. **g** The protein level of Mettl3 and Esr1 in muscle stem/progenitor cells from the concave and convex side of unilateral OS mice. **h** Representative immunofluorescent staining of bilateral paraspinal muscle sections from the unilateral OS group. Green indicates laminin; blue indicates DAPI staining of nuclei. The merged images are shown. Scale bars, 100 μm. **i**, Statistical analysis of the CSA of myofibers from the unilateral OS group. At least 400 fibers were analyzed for each sample. Error bars indicate the standard deviation (*n* = 6). **P < 0.01.

We proceeded to investigate whether unilateral oxidative stress of paraspinal muscle could enhance the propensity for scoliosis in vivo using a bipedal mouse model. H_2_O_2_, known to elevate ROS levels as previously reported^41^, was injected into the left side of the paraspinal muscles, while PBS served as a control and was injected into the right side. This regimen was maintained for 3 weeks, with injections administered twice weekly (Fig. 4a). Two weeks after treatment, spinal alignment was assessed by X-ray, revealing that the group receiving unilateral H_2_O_2_ injections exhibited more severe spinal malformations in both the coronal and sagittal planes compared with the group receiving bilateral PBS injections (Fig. 4b–d). Then, the paraspinal muscles from both sides were collected for further analysis. Both global m^6^A level evaluation and MeRIP–qPCR for target functional modification sites indicated that m^6^A activity was markedly reduced on the concave paraspinal muscle stem/progenitor cells relative to the convex side (Fig. 4e,f). Western blot analyses also demonstrated decreased protein levels of Mettl3 and Esr1 on the concave paraspinal muscle stem/progenitor cells compared with the convex side (Fig. 4g), verifying the suppressed Mettl3–Esr1 axis by accumulated ROS. In addition, the myofiber size on the side injected with H_2_O_2_ (concave side) was found to be smaller than that on the PBS-injected side (convex side) (Fig. 4h,i), indicating impaired paraspinal muscle growth after oxidative stress. Taken together, these findings suggest that unilateral oxidative stress of paraspinal muscle paraspinal muscles can lead to scoliosis in vivo through the ROS–Mettl3–Esr1 axis.

### Betaine could mitigate the differentiation defects of muscle stem/progenitor cells on the concave side

In view of the results that unilateral oxidative stress of paraspinal muscle could lead to scoliosis through the ROS–METTL3–ESR1 axis in an m^6^A-dependent manner, enhancing antioxidant capacity and m^6^A modification levels may counteract the downregulation of ESR1 in muscle stem cells on the concave side, offering a potential therapeutic approach for AIS. Betaine (trimethyl glycine), a stable and nontoxic compound widely utilized in pharmaceuticals, health product research, feed additives and other industries, is recognized for its potent antioxidant properties^30–32^. Furthermore, as a methyl donor, betaine can also markedly enhance m^6^A modification levels^33,34^.

**Fig. 5:**
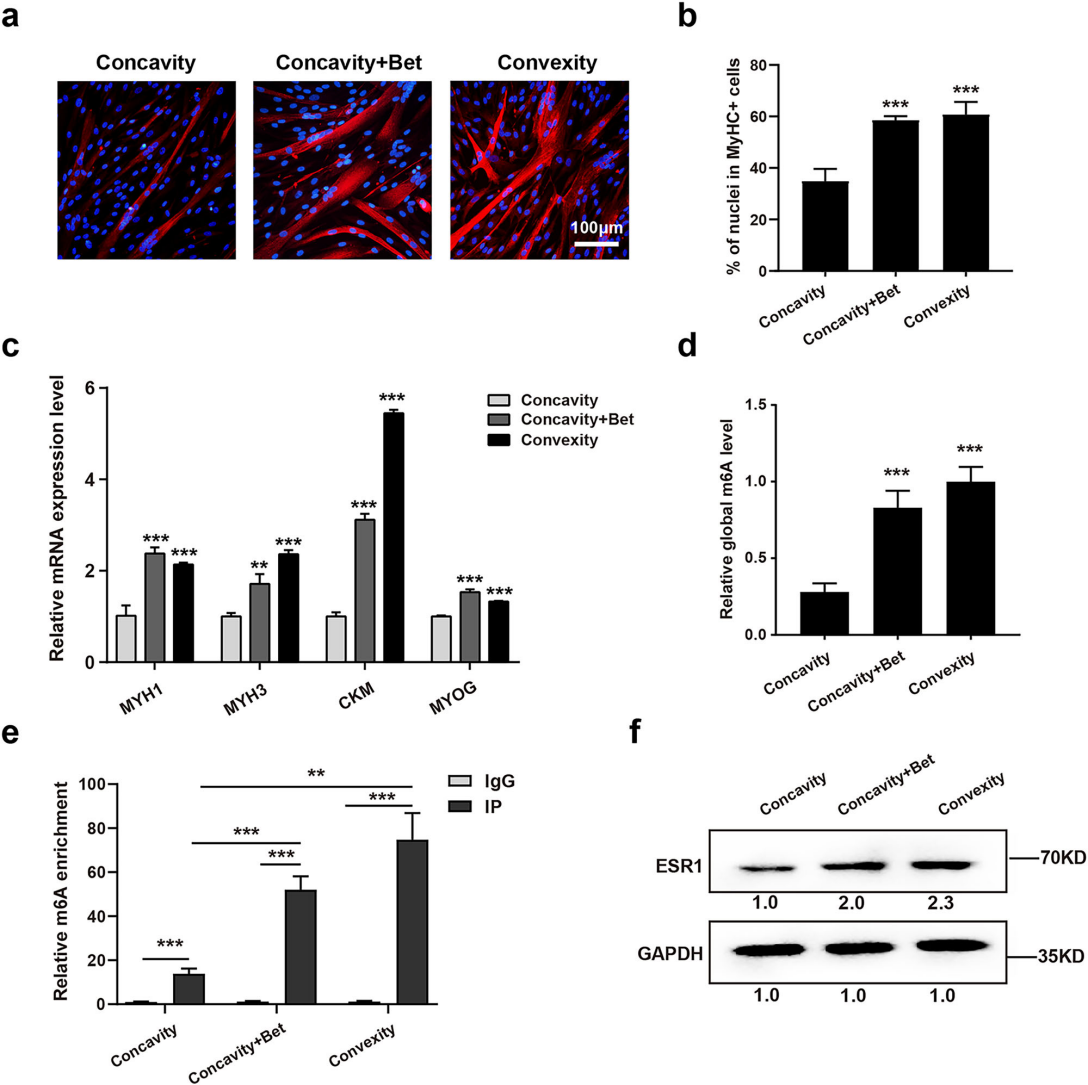
Betaine could mitigate the differentiation defects of muscle stem/progenitor cells on the concave side. **a** Representative immunofluorescent staining of myotubes differentiated from human muscle stem/progenitor cells treated with betaine (Bet). Human muscle stem/progenitor cells were isolated from the convex and concave side of patients with AIS. Red indicates MyHC; blue indicates DAPI staining of nuclei. The merged images are shown. Scale bars, 100 μm. **b** Quantification of percentage of nuclei in MyHC^+^ cells. ***P < 0.001. **c** Relative mRNA expression levels of myogenic differentiation markers. Total RNA was extracted from myotubes differentiated from muscle stem/progenitor cells with different treatments followed by RT–qPCR analysis. *n* = 3. ***P < 0.001. **d** The global content of m^6^A of myotubes differentiated from muscle stem/progenitor cells with different treatments. ***P < 0.001. **e** MeRIP–qPCR results for target m^6^A modification sites in muscle stem/progenitor cells with different treatments. **P < 0.01, ***P < 0.001. **f** The protein expression levels of Esr1 in muscle stem/progenitor cells with different treatments.

Thus, betaine was used to investigate its potential rescue effect on concave paraspinal muscle stem/progenitor cells. As expected, immunofluorescent staining of MyHC showed that betaine mitigated the differentiation defects of human muscle stem/progenitor cells on the concave side (Fig. 5a,b). Consistently, the mRNA expression of *MYH1*, *MYH3*, *CKM* and *MYOG* tested by RT–qPCR showed the same results (Fig. 5c). In addition, both global m^6^A level evaluation and MeRIP–qPCR for target functional modification sites demonstrated an increase in m^6^A activity in these cells following betaine treatment (Fig. 5d,e). Western blot tests also revealed an increase in Esr1 protein levels in concave paraspinal muscle stem/progenitor cells after betaine treatment (Fig. 5f). These results suggest that betaine could serve as a promising therapeutic agent to effectively rescue the differentiation defects of human muscle stem/progenitor cells on the concave side.

### Betaine alleviates the progression of scoliosis in vivo through the ROS–Mettl3–Esr1 axis

Given that betaine can correct the differentiation deficiencies in muscle stem/progenitor cells from the concave-side paraspinal muscle, we investigated its potential as an in vivo treatment for scoliosis.

**Fig. 6:**
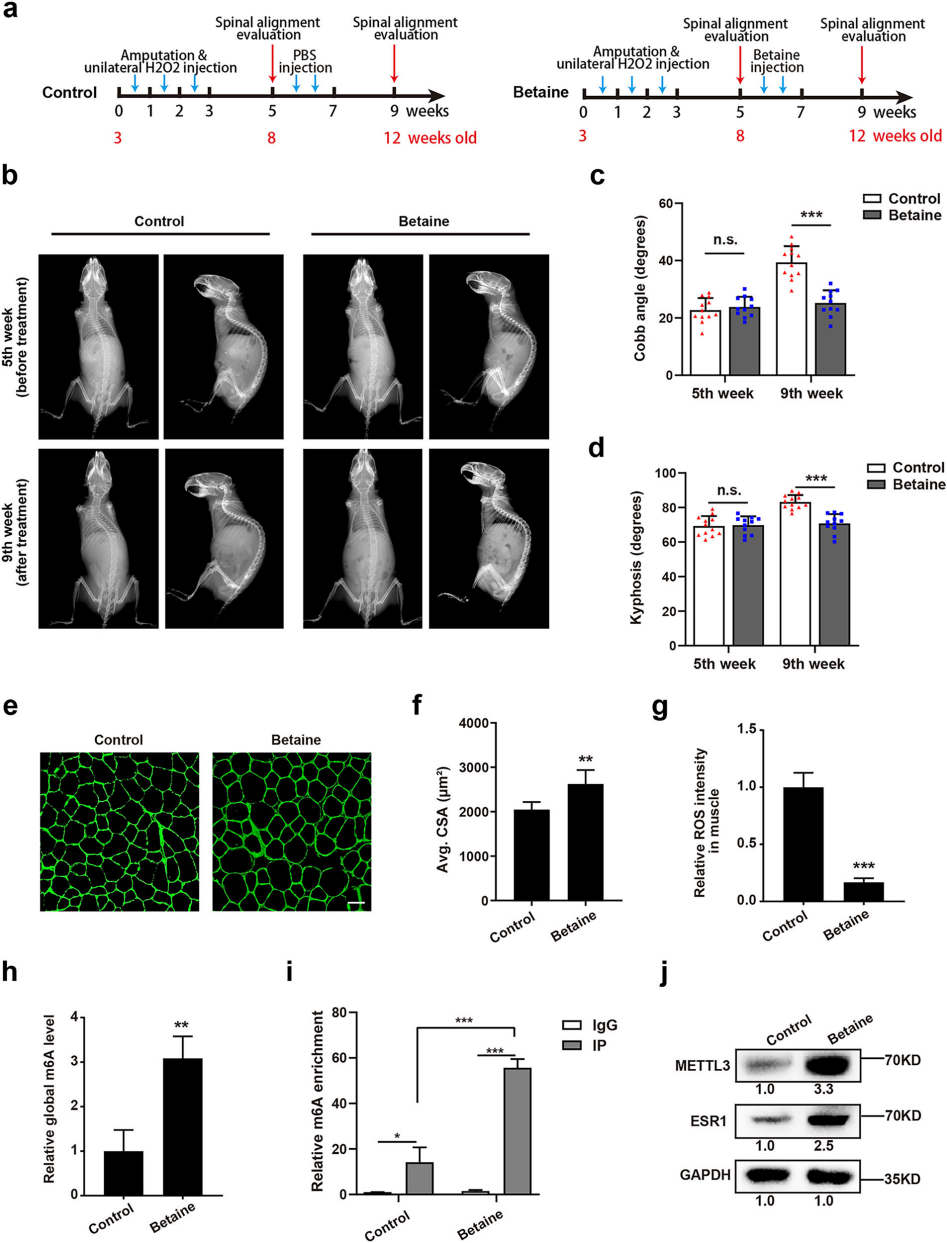
Betaine alleviates the progression of scoliosis in vivo through the ROS–Mettl3–Esr1 axis. **a** Diagram of the betaine rescue strategy in a bipedal mouse model with unilateral oxidative stress. **b** X-ray images for spinal alignment in the control and betaine groups 2 weeks after the last treatment. **c**,**d** Statistical analysis of Cobb angle and kyphosis in coronal and sagittal plane for the control group (*n* = 12) and betaine group (*n* = 11). **P < 0.01, ***P < 0.001; ns indicates no significant changes. **e** Representative immunofluorescent staining of paraspinal muscle sections from the control group and betaine group. Green indicates laminin; blue indicates DAPI staining of nuclei. The merged images are shown. Scale bars, 100 μm. **f** Statistical analysis of the cross-sectional area (CSA) of myofibers in concave paraspinal muscle from the control group and betaine group. At least 400 fibers were analyzed for each sample. Error bars indicate the standard deviation (*n* = 6). **P < 0.01. **g** Relative ROS intensity in concave paraspinal muscles derived from the control group and betaine group. ***P < 0.001. **h** The global content of m^6^A in concave paraspinal muscle stem/progenitor cells from the control group and betaine group. **P < 0.01. **i** MeRIP–qPCR results for target m^6^A modification sites in concave paraspinal muscle stem/progenitor cells from the control group and betaine group. *P < 0.05, ***P < 0.001. **j** The protein expression of Mettl3 and Esr1 in concave paraspinal muscle stem/progenitor cells from the control group and betaine group.

The scoliosis mouse model was first established by unilateral injection of H_2_O_2_ as described in Fig. 4a. Two weeks after H_2_O_2_ injection (8 weeks old), betaine was administered to the concave side twice weekly for 2 weeks, with PBS being injected into the opposite side as a control. A separate control group received bilateral PBS injections twice weekly for the same duration (Fig. 6a). Spinal alignment was evaluated at 5 and 9 weeks after the first injection (Fig. 6a). The X-ray evaluation for spinal alignment indicated that betaine treatment notably ameliorated spinal deformities in both the coronal and sagittal planes compared with data at the fifth and ninth week (Fig. 6b–d). Subsequently, paraspinal muscles from both sides were collected for further analysis at the ninth week. The myofiber size on the concave side in the betaine-injected group was larger compared with that in the control group (Fig. 6e,f). In addition, the ROS intensity was notably reduced on the concave side in the betaine treatment group (approximately 16.7% of that in the control group), indicating the robust antioxidant capacity of betaine in vivo (Fig. 6g). Then, fresh paraspinal muscle stem cells were isolated. Both global m^6^A level evaluation and MeRIP–qPCR for target functional modification site revealed an increase in m6A activity on the concave paraspinal muscle stem cells following betaine treatment (Fig. 6h,i). Furthermore, western blot tests confirmed that protein levels of Mettl3 and Esr1 were also elevated on the concave side after betaine treatment (Fig. 6j). These data demonstrate that betaine could elevate the m^6^A modification levels of *Esr1* and thus upregulate the expression level in muscle stem cells on the concave side. Taken together, betaine could serve as a promising therapeutic agent for the treatment of AIS through the ROS–Mettl3–Esr1 axis.

## Discussion

AIS is a prevalent and unexplained spinal deformity. Among the myriad theories proposed to unravel its pathogenesis, muscle imbalance has emerged as a considerable contender in the etiology of AIS. Our previous research revealed differential *ESR1* expression of paraspinal muscle stem/progenitor cells, which further established a correlation between asymmetric myogenesis and the onset and progression of AIS. Nevertheless, numerous challenges persist in translating these findings into practical applications. Therefore, it is imperative to further investigate the regulatory mechanisms underlying the asymmetric *ESR1* expression to uncover safer and more efficacious treatment strategies for AIS.

In this current study, we found that the expression of *ESR1* of concave paraspinal muscle stem/progenitor cells can be modulated by METTL3-mediated m^6^A modification. High ROS levels in concave paraspinal muscle of patients with AIS decreased the expression of METTL3 and thus inhibited the expression of *ESR1* in an m^6^A-dependent manner. Moreover, improving the antioxidant defenses and m^6^A methylation levels effectively reversed the downregulation of *ESR1* and bolstered the differentiation capacity of muscle stem cells on the concave side. By rectifying the imbalance in ROS intensity and m^6^A levels between bilateral paraspinal muscles using the naturally derived substance betaine, we were able to ameliorate the progression of scoliosis, offering a promising therapeutic strategy for AIS.

A pivotal discovery in our study is the correlation between elevated ROS levels and reduced m^6^A levels on the concave side of the paraspinal muscles. Prior research has shown that oxidative stress exerts a dual effect on skeletal muscle: although moderate levels can be beneficial, excessive ROS can lead to impaired muscle force and muscle atrophy^42–45^. Concurrently, muscle atrophy, a characteristic of the concave side paraspinal muscles in patients with AIS, suggests a potential link between ROS and AIS. Although previous studies have indicated higher ROS levels in patients with AIS compared with controls, the precise role of ROS in the asymmetry of spinal muscles remains elusive^46^. This study reported a notable increase in ROS intensity in the concave-side paraspinal muscles of patients with AIS. To ensure the rigor of our findings, we included an age-matched CS group as a stringent control, which allowed us to isolate the specific impact of ROS on the concave side in the context of AIS.

In tandem with the observed increase in ROS intensity, we also noted a marked decrease in m^6^A levels on the concave side of patients with AIS, and further tests revealed that METTL3 played a key role in this process. The m^6^A modification, orchestrated by METTL3, is essential for maintaining muscle mass and ensuring hypertrophic performance^25^. Moreover, many studies showed that different methylation levels are closely associated with AIS curve severity^47,48^. The concurrent asymmetry in ROS and METTL3 levels warrants deeper exploration. It has been documented that abnormally elevated ROS levels in human epidermal cells and embryonic lung fibroblasts can downregulate the expression of specific genes through METTL3-mediated m^6^A modification^49^. Our study identified a similar regulatory pattern in muscle stem/progenitor cells. These findings suggest that the myogenesis defects observed on the concave side of patients with AIS could be orchestrated by a reduction in METTL3 levels, which is a consequence of the high-ROS environment.

Further results in the current study confirmed the presence of an m^6^A modification site in the *Esr1* gene, with the 2,409-bp region showing functional interaction with Mettl3. The disproportionately high incidence of AIS in females suggests a strong link between estrogen and the condition. Building on our previous work that established a connection between asymmetric *ESR1* expression and the progression of AIS, our current research has uncovered that *ESR1* can be regulated by METTL3, which is influenced by the ROS environment.

However, it should be noted that humans are the only species to develop AIS, and there is still no widely recognized mouse model for scoliosis. We developed mouse scoliosis models according to previous studies^18,35^, and the results indicated that unilateral oxidative stress of paraspinal muscle leads to scoliosis through the ROS–Mettl3–Esr1 axis. In addition, paraspinal muscle samples analyzed in this study were obtained from patients with severe scoliosis (Cobb angle >40°), so caution is warranted when extrapolating these findings to patients with mild scoliosis. This discovery highlights a novel target for correcting imbalances in the paraspinal muscles of patients with AIS, potentially offering a new strategy for therapeutic intervention.

Physical exercise and bracing management were routinely recommended for patients with AIS with a Cobb angle less than 40° (ref. ^50^). However, lack of effective theoretical guidance leads to a variety of treatment modalities, making it challenging to assess their efficacy^51,52^. Moreover, the long and painful treatment courses lead to poor patient compliance^53,54^. The mechanism-independent drug therapy revealed in this study might provide a safer and more practical strategy to treat AIS. Betaine, a naturally occurring and nontoxic compound widely utilized in the pharmaceutical industry, has been recognized as a potent antioxidant and primary source of *S*-adenosylmethionine, the principal methyl group donor for m^6^A mRNA methylation^30,31^. Given these attributes, betaine emerges as an ideal candidate for mitigating the progression of scoliotic curves and could pave the way for the development of effective AIS treatments. The utility, safety and feasibility of betaine in clinical AIS treatment merit further investigation.

## Supplementary information

Supplementary Information

## Data Availability

The authors confirm that all data generated and analyzed during this study are either included in this published article or available from the corresponding authors upon reasonable request.
