# Supplementary material for: The asymmetrical ROS–METTL3–ESR1 axis in paraspinal muscle progenitor cells determines the progression of adolescent idiopathic scoliosis
Source: Exp Mol Med. 2026 Mar 5;58(3):725–38. doi: 10.1038/s12276-026-01658-7 (PMC13049166; doi:10.1038/s12276-026-01658-7)
Supplement: Supplementary file 1 — Supplementary Information [file 12276_2026_1658_MOESM1_ESM.pdf]

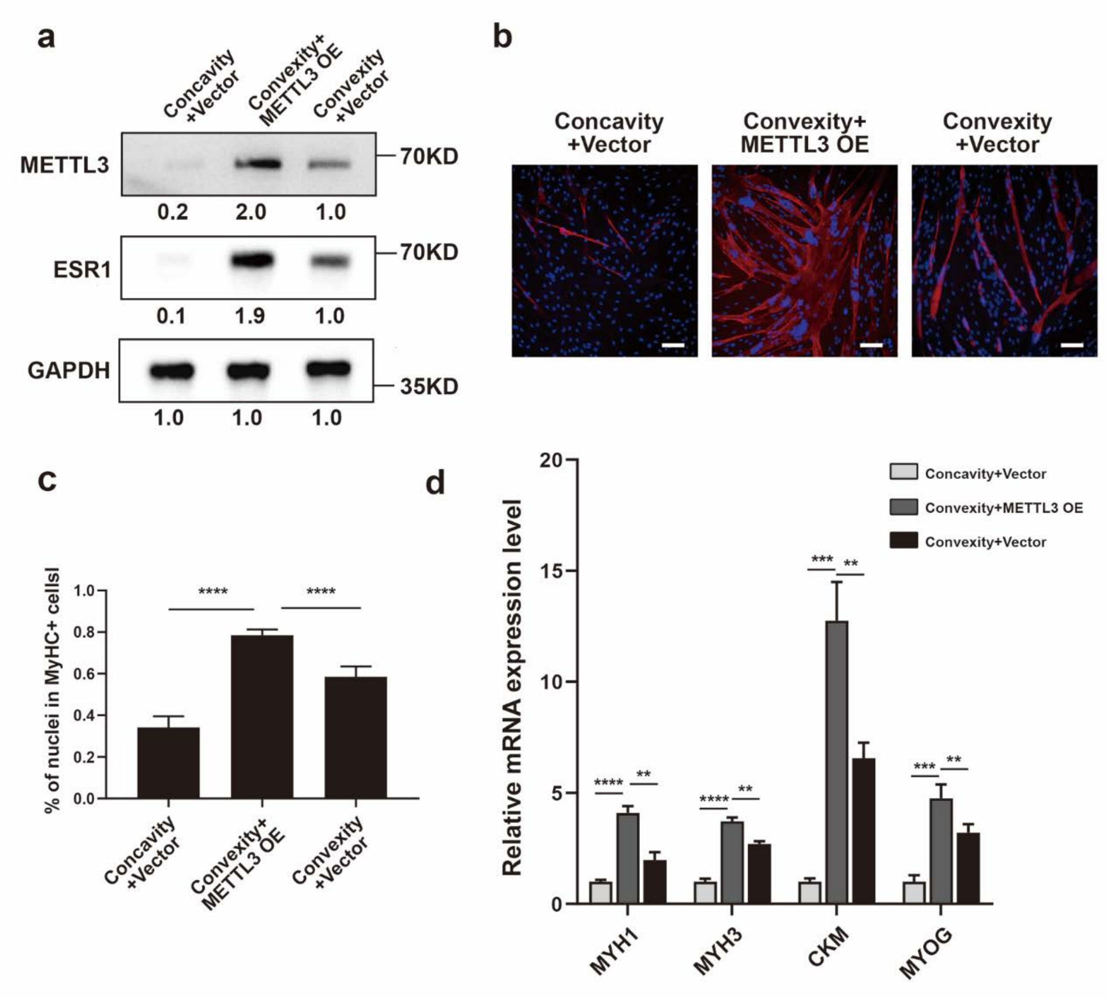

Supplementary Fig. 1 METTL3 overexpression in convex-side progenitors could induce asymmetrical differentiation.

a. The protein level of ESR1 and METTL3 for bilateral muscle stem/progenitor cells transfected with empty plasmid or METTL3 overexpression plasmid. Human muscle progenitors were isolated from convex and concave side of AIS patients. GAPDH was served as control. **b** Representative immunofluorescent staining of myotubes differentiated from bilateral muscle stem/progenitor cells transfected with empty plasmid or METTL3 overexpression plasmid. Human muscle progenitors were isolated from convex and concave side of AIS patients. Red indicated MyHC; blue indicated DAPI staining of nuclei. The merged images were shown. Scale bars: 100 μm. **c** Quantification of percentage of nuclei in MyHC+ cells. ****P < 0.0001. **d** Relative mRNA expression levels of myogenic differentiation markers. Total RNA was extracted from myotubes differentiated from bilateral muscle stem/progenitor cells transfected with empty plasmid or METTL3 overexpression plasmid followed by RT-qPCR analysis. n = 3. **P < 0.01, ***P < 0.001.
